# Editorial: Molecular predictive pathology in gynecologic malignancies

**DOI:** 10.3389/fonc.2023.1301768

**Published:** 2023-10-04

**Authors:** Umberto Malapelle, Stefano Uccella, Sabrina Chiara Cecere, Carmine De Angelis, Pierluigi Giampaolino

**Affiliations:** ^1^ Department of Public Health, University of Naples Federico II, Naples, Italy; ^2^ Department of Obstetrics and Gynecology, University Hospital of Verona, University of Verona, Verona, Italy; ^3^ Department of Urology and Gynecology, Istituto Nazionale Tumori IRCCS Fondazione G. Pascale, Napoli, Italy; ^4^ Department of Clinical Medicine and Surgery, University Federico II, Naples, Italy

**Keywords:** gynecological tumors, molecular biomarkers, liquid biopsy, genomic instability, target drug

Gynecologic malignancies (including endometrial, ovarian, cervical, etc.) represent one of the most common causes of mortality in women (1). The main cause of this phenomenon is related to the absence, except for cervical cancer (2), of valid screening approaches. As a matter of facts, current treatment strategies for advanced stage patients include chemotherapy and radiotherapy. Remarkably, as for other malignancies, giant strides have been made in the field of targeted therapies. Therefore, the identification and correct assessment of predictive biomarkers is pivotal to elect patients for targeted therapies. In this complex scenario, molecular predictive pathology has acquired a key role in the management of these patients (3). The efficacy of Poly (ADP-ribose) Polymerase (PARP) inhibitors (PARPi) in patients harboring genomic alterations in breast cancer (*BRCA*) 1 and 2 genes has been widely demonstrated in high grade serous ovarian carcinoma (HGSOC) (4), and a careful attention has been paid to the role of immune-checkpoint inhibitors (ICIs) in patients harboring a high microsatellite instability (MSI-H) status (5).

In this Special Topic of Frontiers in Oncology, we would like to discuss the methods, findings and prospects of evidence from molecular pathology that will help in the early diagnosis, treatment decision-making, and drug resistance prediction in gynecological malignancies.

Overall, the role of molecular pathology in the management of advanced stage gynecological malignancies has rapidly evolved during the last years. In particular, a number of different genomic alterations have been reported, and may be potential target for personalized therapies (Jiang et al, Tang and Hu) In particular, Jiang et al reported that almost all analyzed patients (94.57%) harbored at least one mutation within *TP53*, *PIK3CA*, *PTEN*, *KRAS*, *BRCA1*, *BRCA2*, *ARID1A*, *KMT2C*, *FGFR2*, and *FGFR3* genes. Interestingly, patients with ovarian cancer showed a high rate of *BRCA1/2* mutations. Of note, patients harboring *TP53*, *PIK3CA*, *PTEN*, and *FGFR3* mutations showed a high tumor mutational burden.

As far as ovarian cancer is concerned, beyond *BRCA1/2* genomic alterations, other potential biomarkers are currently under investigation. Among these, STAT1, STAT4, and STAT6 may be potential targets as proposed by Gong et al. Beyond the predictive role, other biomarkers showed promising results for prognostic purposes. In this setting, Ryu et al. highlighted that the simultaneous expression of β-arrestin and glucorticoid receptor is associated with poor prognosis in ovarian cancer patients. Similarly, Song et al. demonstrated that NCOA5 high expression is associated with disease progression and can be considered as an independent factor affecting the prognosis of ovarian cancer patients. In another experience, Haque et al. showed that VGLL3 mRNA expression was significantly correlated with both advanced tumor stage and poor overall survival. From a diagnostic point of view, Galan et al. demonstrated the role of gangliosides GD2 and GD3 in the diagnosis of ovarian carcinoma in all stages with a high rate of selectivity and specificity.

Considering endometrial carcinoma, Passarelli et al. highlighted the positive predictive role of *PIK3CA* mutations for alpelisib administration. They reported, for the first time, an exceptional response and a good tolerance to alpelisib in a patient with advanced endometrial carcinoma harboring *PIK3CA* mutation.

Molecular pathology may play a crucial role in the diagnostic process, in particular in those morphological trouble cases. In the experience by Lu et al., the Authors highlighted the crucial role of the *COL1A1*–*PDGFB* fusion to refine the diagnosis of rare uterine sarcoma at cervix. Considering cervical cancer, significant advances have been made in the field of treatment. In particular, Li et al. showed that the expression of N-glycopeptide of MASP1, LUM, ATRN, CO8A, CO8B and CO6 may be potential biomarkers for predicting the efficacy of chemotherapy for these patients. In addition, a comprehensive genomic profiling associated with PD-L1 expression may help to select patients for ICIs administration.(Kim et al.) From a prognostic point of view, it has been highlighted the role of RPL24 as a potential biomarker to predict the prognosis of cervical cancer patients and assess chemotherapy efficacy.(Ming et al.)

Finally, an emerging tool for molecular purposes is represented by extracellular vesicles, that have demonstrated their utility as a novel biomarker and therapeutic target. (Wang et al.; Kong et al.)

Overall, this Research Topic has highlighted the recent evidences from molecular pathology that will help in the early diagnosis, treatment decision-making and drug resistance prediction in gynecological malignancies.

Ongoing research is warranted to improve the clinical outcome of these patients.

## Author contributions

UM: Conceptualization, Data curation, Formal Analysis, Funding acquisition, Investigation, Methodology, Project administration, Resources, Software, Supervision, Validation, Visualization, Writing – original draft, Writing – review & editing. SU: Conceptualization, Data curation, Formal Analysis, Funding acquisition, Investigation, Methodology, Project administration, Resources, Software, Supervision, Validation, Visualization, Writing – original draft, Writing – review & editing. SC: Conceptualization, Data curation, Formal Analysis, Funding acquisition, Investigation, Methodology, Project administration, Resources, Software, Supervision, Validation, Visualization, Writing – original draft, Writing – review & editing. CA: Conceptualization, Data curation, Formal Analysis, Funding acquisition, Investigation, Methodology, Project administration, Resources, Software, Supervision, Validation, Visualization, Writing – original draft, Writing – review & editing. PG: Conceptualization, Data curation, Formal Analysis, Funding acquisition, Investigation, Methodology, Project administration, Resources, Software, Supervision, Validation, Visualization, Writing – original draft, Writing – review & editing.

## References

[1] Siegel RL Miller KD Wagle NS Jemal A. Cancer statistics, 2023. CA Cancer J Clin (2023) 73:17–48. doi: 10.3322/caac.21763 36633525

[2] Wang J Bai Z Wang Z Yu C. Comparison of secular trends in cervical cancer mortality in China and the United States: an age-period-cohort analysis. Int J Environ Res Public Health (2016) 13:1148. doi: 10.3390/ijerph13111148 27869688 PMC5129358

[3] Bejar FG Oaknin A Williamson C Mayadev J Peters PN Secord AA. Novel therapies in gynecologic cancer. Am Soc Clin Oncol Educ Book. (2022) 42:1–17. doi: 10.1200/EDBK_351294 35594502

[4] Fong PC Boss DS Yap TA Tutt A Wu P Mergui-Roelvink M. Inhibition of poly(ADP-ribose) polymerase in tumors from BRCA mutation carriers. N Engl J Med (2009) 361:123–34. doi: 10.1056/NEJMoa0900212 19553641

[5] Mirza MR Chase DM Slomovitz BM dePont Christensen R Novák Z Black D. Dostarlimab for primary advanced or recurrent endometrial cancer. N Engl J Med (2023) 388:2145–58. doi: 10.1056/NEJMoa2216334 36972026

